# Fluorescence Confocal Microscopy in Urological Malignancies: Current Applications and Future Perspectives

**DOI:** 10.3390/life13122301

**Published:** 2023-12-05

**Authors:** Luca Ongaro, Giulio Rossin, Arianna Biasatti, Matteo Pacini, Michele Rizzo, Fabio Traunero, Andrea Piasentin, Alessandro Perotti, Carlo Trombetta, Riccardo Bartoletti, Alessandro Zucchi, Alchiede Simonato, Nicola Pavan, Giovanni Liguori, Francesco Claps

**Affiliations:** 1 Urological Clinic, Department of Medicine, Surgery and Health Sciences, University of Trieste, 34149 Trieste, Italy; ongarluc@gmail.com (L.O.); giulio.rossin93@gmail.com (G.R.); arianna.biasatti@gmail.com (A.B.); mik.rizzo@gmail.com (M.R.); fabio.tra92@gmail.com (F.T.); andrea.piasentin23@gmail.com (A.P.); trombcar@units.it (C.T.); gioliguori33@gmail.com (G.L.); 2 Urology Unit, Department of Translational Research and New Technologies in Medicine and Surgery, University of Pisa, 56126 Pisa, Italy; matteopacini93@gmail.com (M.P.); perotti.alessandro@yahoo.it (A.P.); riccardo.bartoletti@unipi.it (R.B.); zucchi.urologia@gmail.com (A.Z.); 3 Urology Clinic, Department of Surgical, Oncological and Stomatological Sciences, University of Palermo, 90127 Palermo, Italy; alchiede@gmail.com (A.S.); nicpavan@gmail.com (N.P.)

**Keywords:** confocal microscopy, prostate neoplasms, bladder neoplasms, ureteral neoplasms, kidney neoplasms, diagnostic performance

## Abstract

Fluorescence confocal microscopy (FCM) represents a novel diagnostic technique able to provide real-time histological images from non-fixed specimens. As a consequence of its recent developments, FCM is gaining growing popularity in urological practice. Nevertheless, evidence is still sparse, and, at the moment, its applications are heterogeneous. We performed a narrative review of the current literature on this topic. Papers were selected from the Pubmed, Embase, and Medline archives. We focused on FCM applications in prostate cancer (PCa), urothelial carcinoma (UC), and renal cell carcinoma (RCC). Articles investigating both office and intraoperative settings were included. The review of the literature showed that FCM displays promising accuracy as compared to conventional histopathology. These results represent significant steps along the path of FCM’s formal validation as an innovative ready-to-use diagnostic support in urological practice. Instant access to a reliable histological evaluation may indeed significantly influence physicians’ decision-making process. In this regard, FCM addresses this still unmet clinical need and introduces intriguing perspectives into future diagnostic pathways. Further studies are required to thoroughly assess the whole potential of this technique.

## 1. Introduction

Fluorescence confocal microscopy (FCM) is an imaging technique that provides real-time digital images of fresh tissue, without the need for further conventional pathology. It allows real-time microscopic examination with the high-resolution visualization of cells and structures.

Confocal microscopy was first described by Marvin Minsky in 1957 [1]. The key to confocal approach is the elimination of out-of-focus light (also known as flare) by scanning a point source of light across the specimen and using a pinhole to eliminate the out-of-focus light from the detector. When compared to a conventional wide field light microscope, the confocal microscope provides an increase in both the maximum lateral resolution (0.5 μm vs. 0.25 μm) and the maximum axial resolution (1.6 μm vs. 0.7 μm) [2].

It can be used in reflectance (RCM) or fluorescence mode (FCM): RCM is based on the reflection of light from different components of cellular structures, while FCM involves the visualisation of fluorophores to characterise cellular details. FCM harnesses external dyes to obtain fluorescence contrast. To date, the most widely used is Acridine Orange, which binds specifically to DNA thus allowing a clear visualization of the nuclei under the fluorescent laser. Images are obtained in a haematoxylin and eosin (H&E) digital staining, which facilitates the interpretation by pathologists and surgeons. CFM has been approved for clinical use in gastroenterology and pulmonology, specifically for the evaluation of Barrett’s oesophagus, pancreaticobiliary diseases, gastric cancer, and other pathological conditions [3,4,5]. It has also been applied in dermatology, where it is currently used to determine positive margins of basal cell and squamous cell carcinoma during Mohs surgery [6].

The use of fluorescence confocal microscopy is also spreading in urological practice. Over the last ten years various applications have been explored in a bid to validate a useful diagnostic tool able to aid both intraoperative decision making and office followup [7,8].

Considering the urothelial carcinoma (UC) scenario, FCM has been investigated in both bladder cancer (BC) and upper-tract urothelial carcinoma (UTUC). Confocal laser endomicroscopy (CLE) is a unique optical imaging technology that can provide real-time and high-resolution imaging of the cellular architecture and the morphology of mucosal lesions. Its use during transurethral resection of bladder tumours (TURBT) or cystoscopy provides the surgeon with useful histological information and represents a promising technique for conservative BC management [9,10,11]. CLE is a reliable and accurate technique in BC diagnosis [12]. Furthermore, CLE can be performed in patients with UTUC during ureteroscopy [13]. In regard to prostatic specimens’ interpretation, CFM has been applied both in the office setting to study biopsy cores as well as intraoperatively to evaluate surgical margins during radical prostatectomy [14,15]. CFM has also been successfully applied for a real-rime diagnosis of renal cell carcinoma (RCC) [16].

Despite its attractiveness, the widespread diffusion of this technique is still experimental. Hence, we aimed to summarize the current evidence to provide the reader with an extensive overview on CFM applications in urology.

## 2. Materials and Methods

A comprehensive database search of the literature produced until October 2023 was performed. Papers were selected from the Pubmed, Embase, and Medline archives using a combination of the following MeSH keywords: “Confocal Microscopy”, “Prostate Neoplasms”, “Urinary Bladder Neoplasms”, “Carcinoma, Transitional Cell”, and “Kidney Neoplasms”. A total of 167 manuscripts were identified. Among them, 22 studies met the inclusion criteria and were then included. Only original papers in the English language were considered. Editorials, comments, case reports, review articles, and conference abstracts were excluded. Diagnostic outcomes included: Sensitivity (Se), Specificity (Sp), Positive Predictive Value (PPV), Negative Predictive Value (NPV). All outcomes’ measures were collected and reported when provided by the authors.

## 3. Results

### 3.1. Bladder Cancer

BC is a heterogeneous disease encompassing non-muscle-invasive (NMIBC) and muscle-invasive BC (MIBC) and entailing very heterogeneous managements and prognoses [17,18,19,20,21]. The results regarding CFM applications in BC detection are reported in Table 1. Beji et al. reported a 48% Se, an 82.7% Sp, a 54.75 PPV, and an 82.3% NPV in their cohort [22]. The CLE capability to distinguish normal mucosa, inflammatory changes, or low-grade UC (LGUC) samples from high-grade (HGUC) has been investigated. Despite the overall 82.3% NPV, the authors stated that currently CLE cannot safely replace traditional histopathology. Lee et al. separately evaluated the CLE ability to differentiate malignant vs. benign tissue, LGUC vs. HGUC, and carcinoma in situ (CIS) vs. inflammatory mucosal changes [23]. CLE was able to differentiate LGUC from HGUC with an overall Se of 94.5%. Moreover, the same authors reported better recurrence-free survival (RFS) outcomes for the CLE cohort at the 25-month followup. Wu et al. reported a total of 6/7 LGUC cases (85.7%) and 8/10 HGUC cases (80.0%) correctly staged with CLE at final pathology [24]. Lucas et al. described a CLE analysis process implemented through a pretrained neural network [25]. Artificial intelligence (AI)-enhanced frame selection provided accuracy rates of 79% in recognizing non-malignant and malignant tissue and 82% between LGUC and HGUC, respectively. In their cohort, Liem et al. reported a sensitivity of 76% and 70% for LGUC and HGUC detection, respectively [26]. Table 1 presents data about CFM applications in the BC spectrum.

### 3.2. Upper Tract Urothelial Cancer

The results regarding CFM applications in UTUC’s detection are reported in Table 2. Sanguedolce et al. reported an overall concordance of 71.4% between in vivo CLE and ureteral biopsy [28]. Freund et al. described the prevalence of CLE features in both LGUC and HGUC in a cohort of 36 patients scheduled for diagnostic ureteroscopy [29]. The outcomes showed an overall 90% Se for endoscopic CLE-based grading. Prata et al. recently proposed a CFM-based technique to assess real-time ureteral margins’ status during open radical cystectomy [30]. CFM reported a NPV of 83.3% compared to the final conventional pathology. Agreement rates between the CFM and frozen section analysis (FSA) were evaluated through Cohen’s Kappa coefficient (k Cohen). In this regard, the authors reported a k Cohen = 0.712 (*p* < 0.001), defined as “good agreement” at provided intervals.

### 3.3. Prostate Cancer

Prostate cancer (PCa) represents a clinical scenario, where novel technologies have the potential to guide a tailored treatment and personalized management [34,35,36]. The results regarding CFM applications for PCa detection are reported in Table 3. Puliatti et al. focused on prostatic biopsies performed on radical prostatectomy (RP) surgical specimens. Substantial overall diagnostic agreement between CFM and the final pathology was reported with a 91% correct diagnosis rate; the Se and Sp rates were 83.3% and 93.5%, respectively [14]. Marenco et al. evaluated transperineal prostate biopsies on biopsy-naive patients [8]. The authors evaluated CFM and H&E stains as the standard of reference in terms of concordance at the biopsy core and ROI level. The K Cohen coefficient was 0.81 for the biopsy core level and 0.69 for the ROI level, respectively. The PPV and NPV were 85.0% and 95.1% at overall biopsy core analysis, respectively, and 83.8% and 85.7% at the ROI level analysis, respectively. Rocco et al. performed an evaluation of periprostatic tissue along the surgical margins during RP [37]. The agreement between CFM and H&E in discriminating between cancerous and noncancerous tissue was 100%. The same authors also provided interesting findings on the prostatic biopsy cores’ specimens [38]. The diagnostic concordance between CFM and H&E for the detection of PCa was impressively satisfactory (k Cohen = 0.84; 0.81–0.88 among four dedicated uro-pathologists) with a 95.1% correct diagnoses rate obtained (range 93.9–96.2). Gobbo et al. recently published a study on prostatic biopsies’ analysis [39] on the same topic [39]. A strong agreement was obtained for the International Society of Urological Pathology (ISUP)/World Health Organization (WHO) grade group I, IV, and V (k Cohen = 0.85). For the remaining non-malignant stains, agreement was nearly complete (k Cohen = 0.81).

### 3.4. Renal Cell Carcinoma

Results regarding CFM applications in renal cell carcinoma cancer (RCC) are shown in Table 4. To date, only three papers have investigated CFM in RC diagnosis. Mir et al. reported a concordance of 100% between ex vivo CFM analysis and definitive H&E assessment [7]. Liu et al. reached an overall 89.2% accuracy rate as compared to H&E-stained samples [40]. We did not find any data describing in vivo applications for RCC.

## 4. Discussion

CFM represents an innovative and attractive tool, able to provide a real-time histological assessment. Despite being still experimental, urological applications are on the rise. Both in vivo and ex vivo experiences have been reported. Regarding CFM in vivo applications, the reports mainly focused on surgical margins’ evaluation and real-time histological grading. The reported diagnostic outcomes were heterogeneous among the included papers. Nevertheless, CFM has shown intriguing results in various areas. 

UC was the most investigated topic. The technique’s applications have been reported for both BC and UTUC. Histological grade assessment represents one of the most investigated topics in the BC setting. In their paper, Chang et al. first proposed diagnostic criteria for BC grading based on CLE features [10]. Cellular, microarchitectural, and vascular characteristics in CFM images were collected and evaluated. The comprehensive evaluation of the histological pattern provided a real-time grading for BC. Interestingly, high interobserver agreement was documented after only short training sessions with optical biopsies’ images. CLE was surprisingly easily performed and interpreted by novice observers. In the same field, Liem et al. [26] compared pure-WLC and WLC/CLE combined images. Concordance with the final histopathology was higher for the WLC/CLE cohort (68.2% vs. 58.5%). Cellular organisation, morphology, and borders’ appearance were reported as the most discriminating features for grade differentiation. Therefore, the authors concluded that CLE might guarantee an additional support to real-time BC diagnosis. The authors also validated the 2013 Chang’s CFM criteria for BC detection [26]. In their study, Lee et al. provided a comparative analysis between CLE features and histological analysis. The results showed relatively good sensitivity and NPV when discriminating between low-grade and high-grade BC [23]. Consistent with the previous data, Wu et al. explored the CLE ability to correctly grade urothelial lesions with comparable results. The overall reported diagnostic outcomes encourage the implementation of CLE in this setting [24]. Nearly real-time characterisation may help optimize decision making in small low-grade lesions, where office fulguration or active surveillance are feasible and safe approaches [41]. With this purpose, Tang et al. presented a first in-office application of CLE during routinary diagnostic cystoscopy [42]: CLE-enhanced WLC may potentially reduce the risk of both over- and under-treatment in selected patients.

Incomplete TURBT represents one of the main concerns in BC operative management [43]. Some reports evaluated CLE’s ability to distinguish between normal urothelial mucosa and cancerous residual tissue [23,25,26]. This potential may be intraoperatively harnessed to assess resection margins’ status, potentially providing survival benefits. Lee et al. reported a recurrence-free survival advantage for the CLE-aided TURBT cohort compared to the WLC-only group [23]. Nevertheless, larger studies with long-term followup are required to definitively assess the actual impact of CLE on RFS. Moreover, CLE could potentially confirm the presence of detrusor muscle (DM) in operative specimens. DM sampling represents a key point in evaluating TURBT’s completeness. CLE-driven real-time confirmation of DM‘s presence in deep resection samples may reduce the risk of incomplete staging at the final histology. As a consequence, a considerable number of repeated TURBTs could be avoided. However, some authors reported daunting results. Beji et al. concluded that the NPV for CLE was not high enough to safely replace conventional pathology for BC [22]. The authors considered their 82% NPV rate unsafe to omit standard histology, since high-grade lesions may potentially evolve into MIBC. Moreover, CLE has shown controversial results in the evaluation of flat urothelial lesions. The diagnosis of CIS remains one of the major issues in everyday urological practice due to the high risk of progression to MIBC [44]. Since distinction of flat BC is often challenging, technological supports have been developed to enhance WLC diagnostic accuracy. In this regard, major international guidelines currently suggest using visual aids such as photodynamic diagnosis (PDD) fluorescence imaging during TURBT, where available [41]. Nevertheless, Liem et al. concluded that flat bladder lesions remain elusive from assessment through optical biopsy support only [26]. Similarly, Lee et al. reported a significantly lower sensitivity and NPV for CLE when applied to flat lesions [23]. This aspect hinders the application of the technique to discriminate CIS from bladder mucosa’s inflammatory changes.

Application of CLE technology to UTUC management has been also described. Currently, European Association of Urology (EAU) guidelines recommend adopting a kidney-sparing surgical approach for low-risk and selected cases of high-risk UTUC [45]. In this setting, CLE may represent a valuable supportive tool to enhance patients’ conservative management. In vivo CLE experiences during ureteroscopies have been reported. As for BC, real-time CLE-based UTUC grade assessment was the most reported outcome. Variable rates have been described for diagnostic outcomes: Sanguedolce et al. reported a relatively low concordance rate between CLE and biopsy at final pathology (71.4%) [28]. Nonetheless, the same authors reported a 100% Se for high-grade lesion detection. Conversely, Freund et al. described a high concordance between CLE and the final histology for both low-grade and high-grade lesions (90% and 86%, respectively) [29]. The main CLE cytological and microarchitectural features have been reported by the same authors. A total of 17 items were identified through CLE image analysis. Moreover, the authors proposed a CLE-based score to simplify the UTUC grading assessment. Nevertheless, the widespread adoption of the UTUC criteria is currently limited by the lack of an external validation. A single ex vivo CLE application for ureteral cancer detection was provided by Prata et al. [30]. The authors described a CFM-based evaluation for ureteral surgical margins’ evaluation during open radical cystectomy. Optical biopsy showed similar diagnostic results to FSA. However, further studies are necessary to fully assess the potential benefits of CFM in this setting.

As previously reported, CFM applications have also been explored in PCa. Both in-office and intraoperative settings have been explored. Notably, the sensitivity and NPV were generally slightly higher for PCa optical biopsies as compared to BC and UTUC. Remarkably, both Marenco and Rocco reported higher NPV for CFM as compared to traditional H&E histological assessments (95.1% and 96.7%, respectively) [8,38]. Both authors evaluated concordance at prostate biopsies for PCa diagnosis. However, the Se did not reach comparably high rates. CFM-aided real-time surgical margins’ assessment during radical prostatectomy has been explored by Rocco et al. [15]: the technique showed high accuracy in discriminating between non-prostatic tissue, benign prostatic tissue, and PCa. Recently, innovative techniques like PSMA-radioguided surgery have been developed to address the same surgical challenge [46,47]: a synergic path could be potentially developed by combining both approaches. Hopefully, new generation technologies may reduce the risk of positive surgical margins during RP: this might provide survival benefits, especially in high-risk patients.

Preliminary results have been reported for CFM application in RCC management. Nevertheless, only scarce data are available. Mir et al. reported a strong correlation with traditional H&E-stained samples regarding cytoarchitectural features [7]. The largest series on RCC has been provided by Liu et al. [40]. The diagnostic outcomes were comparable to CFM applications in other malignancies, with an overall accuracy of 89.2%. At this point, the main CFM achievement in RCC is represented by the relatively good discrimination between normal parenchyma and cancer tissue. Hence, the technique could find an intraoperative role during partial nephrectomies or renal tumour enucleations. Surgical margins could be assessed in both unfixed renal samples as well as in the resection’s bed.

Despite focusing on a promising topic, our review is burdened by some limitations. A technical limitation for CFM is represented by the superficial-only tissue analysis. The estimated scanning depth is around 60 μm, which hinders the evaluation of deeper histological layers [9]. Therefore, real-time staging may be incomplete or undefinable in some settings. Moreover, we found heterogeneous diagnostic outcomes among the included papers. As a consequence, the procedural accuracy could not be clearly assessed. Where provided, Se and NPV rates were very divergent among different series. Se shifts may be justified by the general lack of definitive CFM image validation protocols. Validated CLE features were provided for BC and PCa only [10,39,48]. Nevertheless, all the authors concluded that larger clinical trials are necessary to refine the CFM criteria. In addition, the lack of standardisation for CFM diagnostic criteria leads to non-standardised training programs, producing a further possible bias. Another drawback of our review sits in the overall small number of patients included in each experimental cohort considered. Larger populations and multicentric studies are required to provide high- quality evidence. As a conclusion, the present results are generally too unreliable to recommend CFM as an alternative to conventional pathology. A consensus panel of experts should be established to explore current knowledge gaps and standardize future research on this topic. Moreover, new multicentric trials should be designed to define and improve CFM diagnostic capabilities.

Finally, the role of AI and machine learning (ML) should be further investigated in this field. ML algorithms integrate and analyse large data amounts to enhance the accuracy of a given task. AI technologies have shown multiple intriguing applications in different urological areas [49,50]. Notably, ML algorithms have recently been proposed to enhance WLC image analysis, improving BC detection in complex scenarios [51]. Similarly, deep learning convolutional neural networks may be trained to detect suspicious features in optical biopsies: in this regard, some successful applications have already been described in different medical disciplines [52,53]. Regarding the urological field, Lucas et al. provided a first preliminary insight of an AI-enhanced workflow [25]. The authors demonstrated the feasibility of AI-assisted CLE for BC, providing similar accuracy rates for BC grading in comparison with real-time WLC. Despite these promising results, the role of AI-assisted CFM is still under-investigated in urological malignancies. Hence the creation of large datasets of representative CLE images should be encouraged. Free access to wide images’ repositories might be helpful to thoroughly explore the real potential of ML in CFM.

## 5. Conclusions

CFM represents a feasible but under-explored technique. Despite CFM being still experimental, the analysis of the current literature highlighted interesting results as well as several intriguing perspectives.

Today, novel cutting-edge technologies have been proposed in multiple urological fields: for instance, even though recently developed, PSMA-radioguided surgery might dramatically change PCa management in the next future. On the other hand, fluorescence-guided technologies are already routinely employed to enhance BC detection at the time of TURBT [54]. Likewise, CFM might be included as part of a multimodal surgical strategy alongside with these innovative procedures. To date, successful attempts to combine fluorescence imaging and optical biopsies have been reported for BC: Gladkova et al. first described the combination of fluorescence cystoscopy and cross polarization optical coherence tomography in 2013 [55]. More recently, Marien et al. proposed the combination of CLE and PDD to enhance BC detection [27]. Therefore, optical biopsies may contribute to the ongoing paradigm shift towards precision surgery: in particular, CFM-driven real-time assessment of excisional surgical margins might provide potential survival improvements.

To conclude, despite being at an early stage, CFM has shown multiple attractive insights for urology. CFM applications are growing, but its routine use remains limited in everyday clinical practice. Further studies are pending to thoroughly explore the full potential of this technique.

## Figures and Tables

**Table 1:** Confocal microscopy in BC.

Author	Year	Pat. (n.)	Setting	CFM System	Procedure	Se. (%)	Sp. (%)	PPV (%)	NPV (%)	Main Outcomes
Beji [22]	2020	12	In vivo	Cellvizio	TURB	48.0 (LGUC vs. HGUC)	82.7 (LGUC vs. HGUC)	54.7 (LGUC vs. HGUC)	82.3 (LGUC vs. HGUC)	NPV for CLE was inadequate to safely replace histopathological assessment for HGUC diagnosis.
Lee [23]	2019	75	In vivo	Cellvizio	TURB	91.7 (mal. vs. ben.) 94.5 (LGUC vs. HGUC) 71.4 (CIS vs. IT)	73.9 (mal. vs. ben.) 66.7 (LGUC vs. HGUC) 81.3 (CIS vs. IT)	93.6 (mal. vs. ben.) 89.7 (LGUC vs. HGUC) 83.3 (CIS vs. IT)	68.0 (mal. vs. ben.) 80.0 (LGUC vs. HGUC) 68.4 (CIS vs. IT)	CLE represents a promising technology to provide real-time reliable diagnosis and grading of UC. Moreover, it might improve RFS.
Wu [24]	2019	21	In vivo	Cellvizio	TURB/DC	NR	NR	NR	NR	The CLE accuracy related to the final biopsy histopathology was 81.0%. A total of six LGUC cases (85.7%) and eight HGBC cases (80.0%) were correctly staged though CLE images.
Lucas [25]	2019	53	In vivo	Cellvizio + AI- image analysis	TURB	NR	NR	NR	NR	CLE accuracy regarding malignant vs. benign tissue distinction was 79%, while the HGUC vs. LGUC differentiation accuracy was 82%.
Liem [26]	2018	53	In vivo	Cellvizio	TURB	76.0 (LGUC) vs. 70.0 (HGUC)	76.0 (LGUC) vs. 69.0 (HGUC)	NR	NR	Concordance between CLE-based classification and final histopathology was found in 19 LGUC cases (76%), 19 HGUC cases (70%), and 4 benign lesion cases (29%).
Marien [27]	2017	9	Ex vivo	Cellvizio	TURB	80.0	100.0	NR	NR	CLE images from seven out of nine patients clearly showed cytoplasm of suspect cells and nuclei.
Chang [10]	2013	31	Ex vivo	NR	TURB	50.0 (LGUC) vs. 75.0 (HGUC)	94.0 (LGUC) vs. 64.0 (HGUC)	NR	NR	Novice CLE observers achieved a diagnostic accuracy comparable to WLC-images-only observation after a short training. An expert CLE observer achieved higher accuracy rates compared to WLC-image-only analysis.

Abbreviations are as follows: Pat. = patients; CFM = confocal microscopy; Se. = sensitivity; Sp. = specificity; PPV = positive predictive value; NPV = negative predictive value; TURB = transurethral resection of the bladder; CLE = confocal laser endomicroscopy; LGUC = low-grade urothelial cancer; HGUC = high-grade urothelial cancer; CIS = carcinoma in situ; mal. = malignant; ben. = benign; IT = inflammatory tissue; AI = artificial intelligence; RFS = recurrence free survival; WLC = white light cystoscopy; NR = not reported.

**Table 2:** Confocal microscopy in UTUC.

Author	Year	Pat. (n.)	Setting	CFM System	Surgery	Se. (%)	Sp. (%)	PPV (%)	NPV (%)	Main Outcomes
Prata [30]	2023	46	Ex vivo	VivaScope 2500	ORC	53.8 (vs. H&E)	90.9 (vs. H&E)	90.9 (vs. H&E)	83.3 (vs. H&E)	CFM showed similar results compared to frozen section analysis for ureteral margins evaluation.
Sanguedolce [28]	2021	7	In vivo	Cellvizio	URS	71.4 (total) 100.0 (HG lesions)	57.1 (for HG lesions only)	NR	NR	Real time concordance with definitive histology in UTUC biopsy: 71.4% (5/7 cases)
Freund [29]	2019	36	In vivo	Cellvizio	URS	90.0	86.0	93.0	80.0	CLE correctly assessed histopathological grading in 26 low-grade UTUCs (90%) and in 12 high-grade UTUCs (86%).
Breda [31]	2017	14	In vivo	Cellvizio	f-URS	NR	NR	NR	NR	Correspondence between CLE images and final histopathological resulted in 7/7 low-grade UTUC (100%), 5/6 high-grade UTUC (83%), 1/1 CIS (100%).
Villa [32]	2016	11	In vivo	Cellvizio	f-URS	NR	NR	NR	NR	CLE allows clear recognition of UTUC histological features.
Bui [33]	2015	14	In vivo	Cellvizio	f-URS	NR	NR	NR	NR	CLE provided images of papillary structures, fibrovascular stalks, and pleomorphism. Lamina propria was identified in normal areas.

Abbreviations are as follows: UTUC = upper tract urothelial cancer; pat. = patients; CFM = confocal microscopy; Se. = sensitivity; Sp. = specificity; PPV = positive predictive value; NPV = negative predictive value; H&E = haematoxylin and eosin; CLE = confocal laser endomicroscopy; ORC = open radical cystectomy; URS = ureteroscopy; f-URS = flexible ureteroscopy; HG = high-grade; NR = not reported.

**Table 3:** Confocal microscopy in PCa.

Author	Year	Pat. (n.)	Setting	CFM System	Se. (%)	Sp. (%)	PPV (%)	NPV (%)	Main Outcomes
Gobbo [39]	2023	NR (75 biopsy slides)	Biopsy	VivaScope	NR	NR	NR	NR	Almost complete agreement was obtained for ISUP/WHO grade group I, IV, and V (k = 0.85). For the remaining noncancer stains, agreement was nearly complete (k = 0.81).
Marenco [8]	2020	57 biopsy-naive men	Biopsy	VivaScope	NR	NR	85.0 (biopsy core) 83.8 (ROI level)	95.1 (biopsy core) 85.7 (ROI level)	CFM and H&E concordance was evaluated on the biopsy core and ROI level; Cohen’s k for agreement between the techniques was 0.81 for the biopsy core level and 0.69 for the ROI level. The PPV and NPV were high at biopsy core and ROI levels.
Rocco [15]	2020	20	Surgical margins (periprostatic tissue) during RP	VivaScope	NR	NR	NR	NR	CFM diagnostic performance in distinguishing between non-prostatic tissue, benign prostatic tissue, and PCa was high; CFM demonstrated almost perfect agreement with H&E in distinguishing all tissue types.
Rocco [38]	2020	54	Biopsy	Vivascope	86.3	97.2	88.5	96.7	The diagnostic agreement between CFM and H&E for the detection of PCa was high (k = 0.84; 0.81–0.88 among four pathologists) with 95.1% correct diagnosis obtained (range 93.9–96.2).
Rocco [37]	2020	8	Surgical margins (periprostatic tissue) during RP	Vivascope	NR	NR	NR	NR	7/8 patients had overall negative SM in the sampled areas. The agreement between CFM and H&E in regard to the discrimination between cancerous and noncancerous tissue was 100%.
Puliatti [14]	2019	13	Biopsy (on RP surgical specimen)	VivaScope	83.3	93.5	NR	NR	The overall diagnostic agreement between CFM and histopathological diagnoses was substantial with 91% correct diagnosis and an AUC of 0.884 (95% CI 0.840–0.920).

Abbreviations are as follows: Pat. = patients; CFM = confocal microscopy; RP = radical prostatectomy; SM = surgical margins; PCa = prostate cancer; ROI = region of interest; H&E = haematoxylin and eosin; AUC = area under the curve; k = Cohen statistic coefficient; PPV = positive predictive value; NPV = negative predictive value; Se. = sensitivity; Sp. = specificity; NR = not reported.

**Table 4:** Confocal microscopy in RCC.

Author	Year	Pat. (n.)	Setting	CFM System	Se. (%)	Sp. (%)	PPV (%)	NPV (%)	Main Outcomes
Mir [7]	2020	4	Ex vivo	VivaScope 2500	NR	NR	NR	NR	Neoplastic and noncancer tissues were both detected in 100% of cases through CFM images analysis (one oncocytoma and three RCC). CFM images showed strong overlapping with traditional H&E-stained samples regarding cytoarchitectural features.
Liu [40]	2016	19	Ex vivo	VR-SIM	79.2	95.1	82.6	90.7	CFM diagnostical outcomes were compared to traditional H&E staining; final accuracy was 89.2%.
Su [16]	2015	20	Ex vivo	Cellvizio	NR	NR	NR	NR	CLE imaging properly evaluates normal renal parenchymal features. It allows a rapid distinction between cancer and normal tissue, as well as the possibility to distinguish between benign and malignant ones. Enhanced CLE images resolution was provided by topical fluorescein rather than by IV route administration.

Abbreviations are as follows: Pat. = patients; CFM = confocal microscopy; RCC = renal cell carcinoma, H&E = haematoxylin and eosin, PPV = positive predictive value; NPV = negative predictive value, IV = intravenous.

## Data Availability

Not applicable.
